# Wearisome Days

**Published:** 1890-06-07

**Authors:** 


### Words of Consolation.

### WEARISOME DAYS.

After a night spent in tossing from side to side in weariness and pain you begin another day with a little feverish strength, which is soon spent, or else with a feeling of languor and exhaustion which unfits you for the duties which lie before you. The necessary washing and dressing, which is really a refreshment, seems beforehand to be more than you can bear. Some friends have promised to call on you, and you are sure they will all come together to-day, and wear you out with talking. You fret yourself with the question whether or not you shall refuse to see them. If you say no they will be hurt, if you let them come you will sink under the infliction. If only some one would decide for you in all such cases, and tell you what you ought to do and what would be best for you! This uncertainty is a keen part of your trials; you dislike the worry and doubt as to what is your duty. You really want to do the right thing, but cannot find out how far you are overtaxing your strength, or, on the other hand, to what extent you are indulging and sparing yourself, when you ought to be taking up your cross and denying your wishes. Perhaps a few rules may be of some assistance in your perplexity.

I. Ask God to direct and rule your heart in all things, and guide you to choose those subjects for thought which will prepare you for future usefulness in the world, should He see fitting to restore you to health.

II. Vary your employments, and never continue even an amusement too long, but leave off as soon as you feel the least fatigue.

III. Have some employment which is purely mechanical constantly at hand, so that mind and body may be amused and not wearied.

IV. Do not be afraid of taking intervals of sheer amusement or perfect rest; they are necessary to restore your spent strength.

V. Do not fill your mind with light reading merely, for any kind of literature which expands the mind makes it stronger, and enables it to sympathise more with others, or hold more communion with God. Generally speaking, tales, and especially novels, are ill-adapted for the reading of sick persons. They take possession of the mind, and haunt it day and night, doing the weak and nervous real harm. To them the story assumes an actual form of truth and life, and if it be tragic they suffer as the characters suffer. Add to this, that if they can read but little it is better to read what will tend to help them forward in the right way, which leadeth into Everlasting Life. Voyages and travels contain enough of interest to take the reader into new scenes, and thus draw his thoughts from himself and his usual train of ideas.

But some sick persons have no leisure—their daily duties, small though they be, take all their strength. For them their strength is to sit still. Let them be very thankful that God has set before them a plain duty, that of giving up to Him their wills. To such we say, Offer up in the morning all your time and strength to God, asking Him to show you hour by hour what He would have you to do, and to give you strength and a willing mind to do it. Ask Him to quiet and strengthen you to go patiently and meekly through the fatigues, as well as the events of the day, and having done so, try and remember as time passes that He is very near to you, and at every fresh need do not fail to speak to Him.

Do not yield to the temptation of looking at everything at once, as if everything would happen at once, and all the events of the day be crowded into an hour. Do not thus forecast evil. Your business is only with the present; leave the future in His hands, who will be sure to do the best, the very best, for you.

